# Targeting DNA repair by coDbait enhances melanoma targeted radionuclide therapy

**DOI:** 10.18632/oncotarget.7340

**Published:** 2016-02-12

**Authors:** Claire Viallard, Jean-Michel Chezal, Florence Mishellany, Isabelle Ranchon-Cole, Bruno Pereira, Aurélie Herbette, Sophie Besse, Zied Boudhraa, Nathalie Jacquemot, Anne Cayre, Elisabeth Miot-Noirault, Jian-Sheng Sun, Marie Dutreix, Françoise Degoul

**Affiliations:** ^1^ Clermont Université, Université d'Auvergne, Imagerie Moléculaire et Thérapie Vectorisée, BP 10448, F-63000 Clermont-Ferrand, France; ^2^ Inserm, U 990, F-63000 Clermont-Ferrand, France; ^3^ Anatomopathology Department, Centre Jean Perrin, Comprehensive Cancer Center, 63011 Clermont-Ferrand, France; ^4^ Clermont Université, Université d'Auvergne, UFR Pharmacie Laboratoire de Biophysique Neurosensorielle, Inserm U 1107, F-63001 Clermont-Ferrand, France; ^5^ DRCI, CHU, 63003 Clermont-Ferrand, France; ^6^ CNRS-UMR3347, INSERMU1021, Institut Curie, Université Paris Sud, Bat 110, Centre Universitaire 91405 Orsay, Cedex, France; ^7^ DNA Therapeutics, SA, 91058 Evry Cedex, France

**Keywords:** targeted radionuclide therapy, melanoma, coDbait, DNA repair

## Abstract

Radiolabelled melanin ligands offer an interesting strategy for the treatment of disseminated pigmented melanoma. One of these molecules, ICF01012 labelled with iodine 131, induced a significant slowing of melanoma growth. Here, we have explored the combination of [^131^I]ICF01012 with coDbait, a DNA repair inhibitor, to overcome melanoma radioresistance and increase targeted radionuclide therapy (TRT) efficacy. In human SK-Mel 3 melanoma xenograft, the addition of coDbait had a synergistic effect on tumor growth and median survival. The anti-tumor effect was additive in murine syngeneic B16Bl6 model whereas coDbait combination with [^131^I]ICF01012 did not increase TRT side effects in secondary pigmented tissues (e.g. hair follicles, eyes). Our results confirm that DNA lesions induced by TRT were not enhanced with coDbait association but, the presence of micronuclei and cell cycle blockade in tumor shows that coDbait acts by interrupting or delaying DNA repair. In this study, we demonstrate for the first time, the usefulness of DNA repair traps in the context of targeted radionuclide therapy.

## INTRODUCTION

Melanoma caused 55 000 deaths in 2012, accounting for 0.7% of all deaths from cancer [1]. A specific trait of cutaneous melanoma is the presence of melanins, a biopigment produced by melanocytes to protect skin from UV radiation. These polymers can serve as a melanoma-specific target through the use of melanin-specific ligands such as antibodies [2] or small molecules of the arylcarboxamide family [3]. In preclinical models, different arylcarboxamide derivatives (ICF01012; MIP-1145) labeled with a $\bar{\beta }$ emitting radionuclide (iodine-131, *t*_1/2_ = 8.02 d) induced significant slowing of tumor growth [4–7]. In a first human study, an MIP-1145 analogue, [^131^I]BA52, was associated with a 2-year survival in 3/5 patients with metastatic melanoma [8]. This study is important since it underlines the safety and efficacy of melanin-TRT against disseminated melanomas but also the need to select patients with pigmented lesions. Indeed, melanins are only present in 32–60% of analyzed metastases as demonstrated by the melanin radiotracer [^123^I]BZA_2_ in a SPECT-CT imaging study [9]. Also, we showed a clear correlation between melanin content and melanin-TRT efficacy in preclinical models [4, 10]. The dosimetry is obviously a major point for successful radiotherapy. However, the maximum administrable dose is limited due to potential adverse effects on retina pointed out in highly pigmented mice [6], but not yet reported in humans [8]. A way to amplify the efficacy of radiation without increasing the dose is to use radiosensitizers. The radioresistance of melanoma [11] may stem from the presence of melanins acting as a free-radical shield around the melanoma cell nucleus. Melanins do exert a radioprotective role by quenching free electrons and free radicals generated from water hydrolysis or by scattering Compton effects [12]. Furthermore, melanoma presents a high DNA instability counterbalanced by efficient repair mechanisms [13–15], which contributes to radioresistance [16]. Therefore, modifying the DNA repair system is of interest to radiosensitize melanoma. A small DNA molecule, called coDbait (Supplementary Figure 1A), possesses such properties. coDbait mimics a DNA double-strand breaks to trap repairing enzymes and disorganizes the DNA repair system [17–19]. It has been tested on different tumors in combination with both external beam radiotherapy (EBR) [20, 21] and chemotherapy [22]. Administered in subcutaneous, coDbait did not induce toxicity in rats or monkeys [23].

To date, no study has investigated the combination of coDbait with internal radiotherapy. Therefore, the ability of coDbait to radiosensitize melanomas to [^131^I]ICF01012 (Supplementary Figure 1B) was tested in syngeneic murine B16Bl6 and human xenograft SK-Mel 3 in which we previously studied the effect of TRT [4, 10]. We also evaluated the side effects the B16Bl6 model and mechanisms underlying TRT ± coDbait in both melanomas.

## RESULTS

### CoDbait treatment plus TRT strongly slowed tumor growth and increased survival in the syngeneic B16Bl6 model by disturbing DNA repair

**Figure 1:**
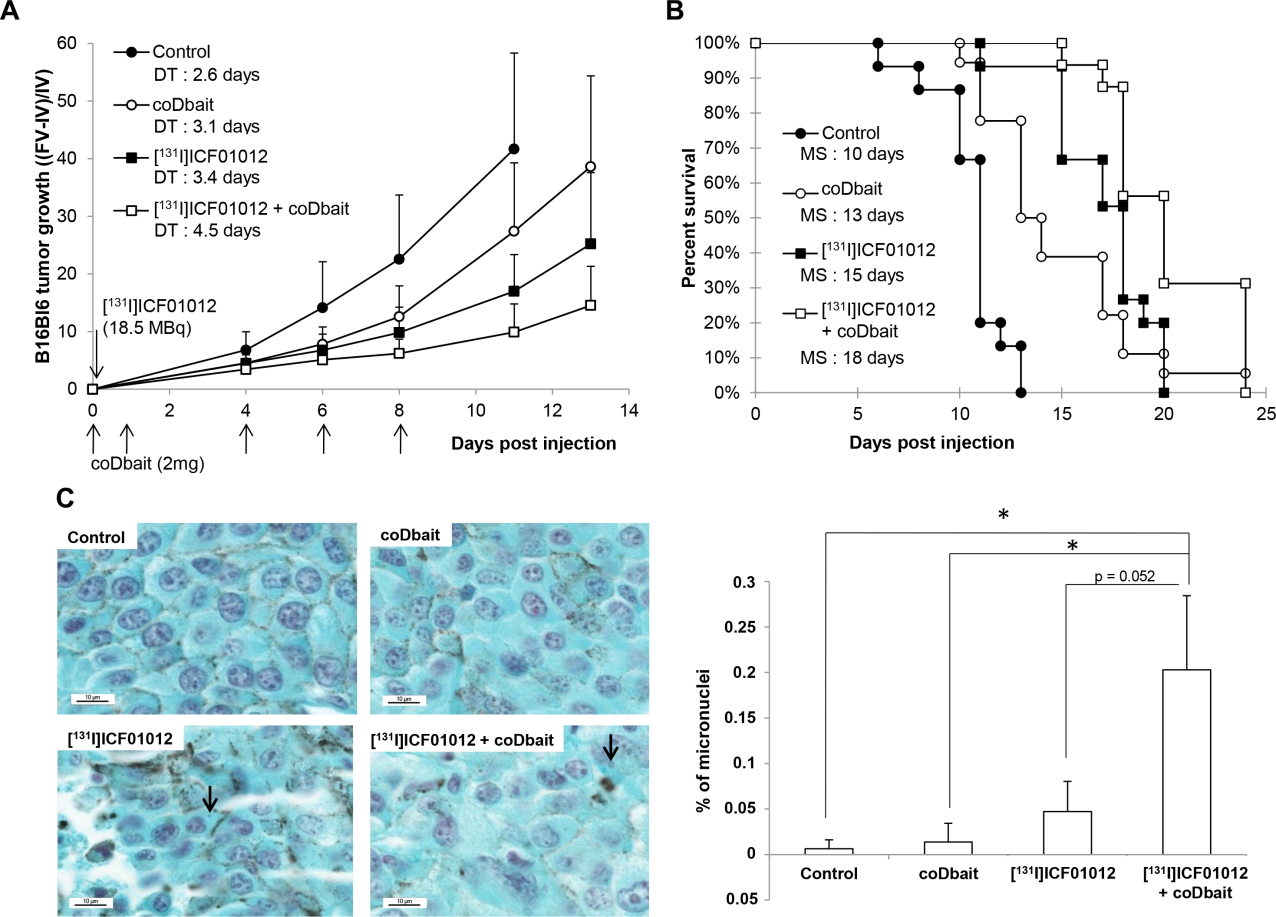
Effect of coDbait added to [^131^I]ICF01012 TRT on tumor growth and median survival in B16Bl6 model (*n* = 20/group) (**A**) The tumor growth was calculated with the ratio of final volume (FV) minus initial volume (IV) to initial volume (IV). The doubling time (DT) was calculated with the exponential value of tumor volume curves. (**B**) Survival curves were established as the percentage of animals that remained in each group (*n* = 20/group) at indicated times (**C**) The number of the cells presenting micronuclei in the cytoplasm (black arrows) in non-necrotic and proliferative areas was increased in [^131^I]ICF01012 TRT group compared to control and coDbait, this was further enhanced by coDbait addition (*n* = 4 /group; 300000 cells counted). *p < 0.05.

The administration of coDbait (5 sessions of 2 mg) during the first week following TRT significantly improved [^131^I]ICF01012 efficacy (Figure 1). [^131^I]ICF01012 + coDbait significantly slowed B16Bl6 growth, the tumor doubling time (DT) being extended by 170% compared with control. Combined treatment was significantly better than either single treatment (Figure 1A) (PHt, *p* < 0.001 compared with control and coDbait groups, *p* = 0.04 compared with [^131^I]ICF01012 group). The evolution of tumor growth using the random effects model demonstrated the additional effects of both molecules (Supplementary Table 1A). Likewise, median survival increased significantly for mice receiving [^131^I]ICF01012 + coDbait treatment (18 days) compared with control (10 days) (Cox, *p* < 0.001) and the single-treatment groups (13 and 15 days for coDbait and [^131^I]ICF01012, respectively) (Cox, *p* = 0.001 and *p* = 0.05, respectively) (Figure 1B). Decrease of lung metastasis number following TRT was not further improved by addition of coDbait (Supplementary Figure 2A) nor was the VEGF tumor content (Supplementary Figure 2B).

Histology analyses were performed on B16Bl6 tumors removed 24 h (2 mg of coDbait) or 10 days (10 mg of coDbait) after irradiation (18.5 MBq). Morphological alterations following TRT (Supplementary Figure 2C) were not increased with coDbait. At 24 h, the mitotic index decreased similarly in TRT and TRT + coDbait (mean: 14 and 13 mitoses per 10 high fields) compared with control and coDbait tumors (mean: > 20 mitoses per 10 high fields). Interestingly, 24 h post-TRT, necrosis was significantly increased (PHt, *p* < 0.001) in the combined treatment group compared with the others (Supplementary Figure 2D). Furthermore, ten days after the treatments, the number of micronuclei was significantly higher in tumors receiving TRT + coDbait (Figure 1C).

### CoDbait treatment plus TRT did not worsen [^131^I]ICF01012 toxicity

**Figure 2:**
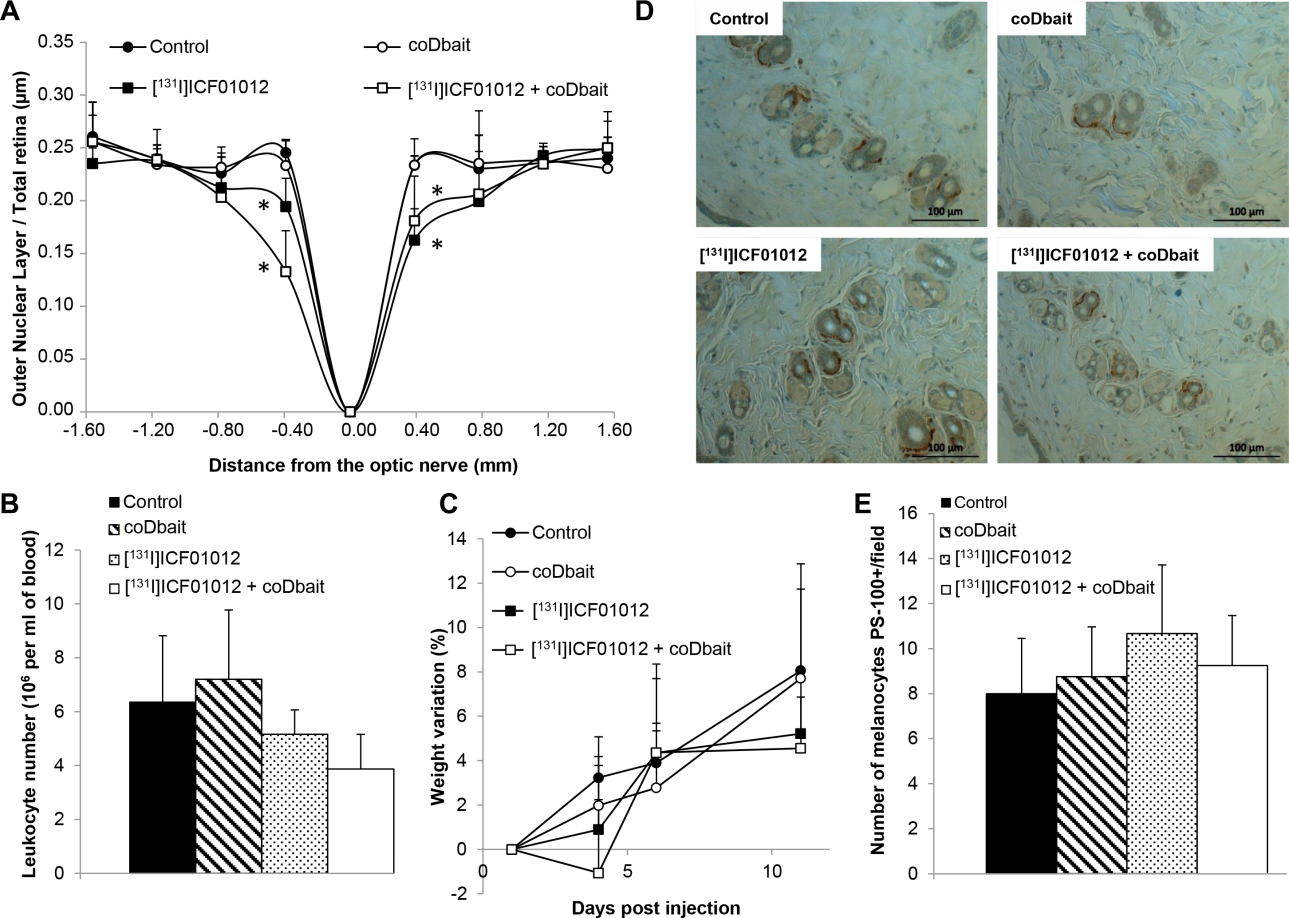
Side effects associated with treatments in pigmented C57Bl6 mice (**A**) Evaluation of [^131^I]ICF01012 + coDbait toxicity on mice retina 10 days post irradiation (*n* = 4 per group). The significant ratio decrease observed following [^131^I]ICF01012 around the optic nerve was not further increased with coDbait addition. (**B**) White blood cell quantification 10 days post irradiation showed a non-significant decrease in irradiated mice. (**C**) % variation from initial values showed no statistically significant weight loss in the four groups. (**D**) Evaluation of [^131^I]ICF01012 radiotoxicity on skin by melanocyte-specific staining of PS100 protein. Representative histological sections of mice skins from the different groups showed a similar number of melanocytes in the hair follicles. (**E**) Quantification of PS-100 stained cells showed no statistically variation of the hair follicle melanocytes. *p < 0.05.

As [^131^I]ICF01012 targets pigmented organs, toxicity effects were studied in eyes and skin. As no histological damage was detected on the ciliary body and choroid structures [6], we focused the analyses on retinal changes, specifically in the outer nuclear layer containing photoreceptors and retinal pigmentary epithelium. Compared with the control group, we observed a significant decrease in retina thickness immediately around the optic nerve area 10 days following irradiation in mice receiving [^131^I]ICF01012 (REM, *p* < 0.001) or [^131^I]ICF01012 + coDbait (REM, *p* < 0.001). However, the difference between the two irradiated groups was non-significant (Figure 2A). The reported leukocyte decrease (Figure 2B) and slight weight loss (Figure 2C) induced by TRT were not modified by the addition of coDbait. These results suggest that coDbait addition to TRT did not increase TRT adverse effects or induce specific toxicity. We evaluated the impact of the combined treatments in hair follicle melanocytes revealed by counting PS100-labelled cells (Figure 2D). The number of melanocytes was identical in controls, single and associated treatment groups (3 to 5/fields, 10 fields counted) (Figure 2E).

### Combination with coDbait significantly enhanced [^131^I]ICF01012 radiotherapy efficacy in SK-Mel 3 human melanomas

**Figure 3:**
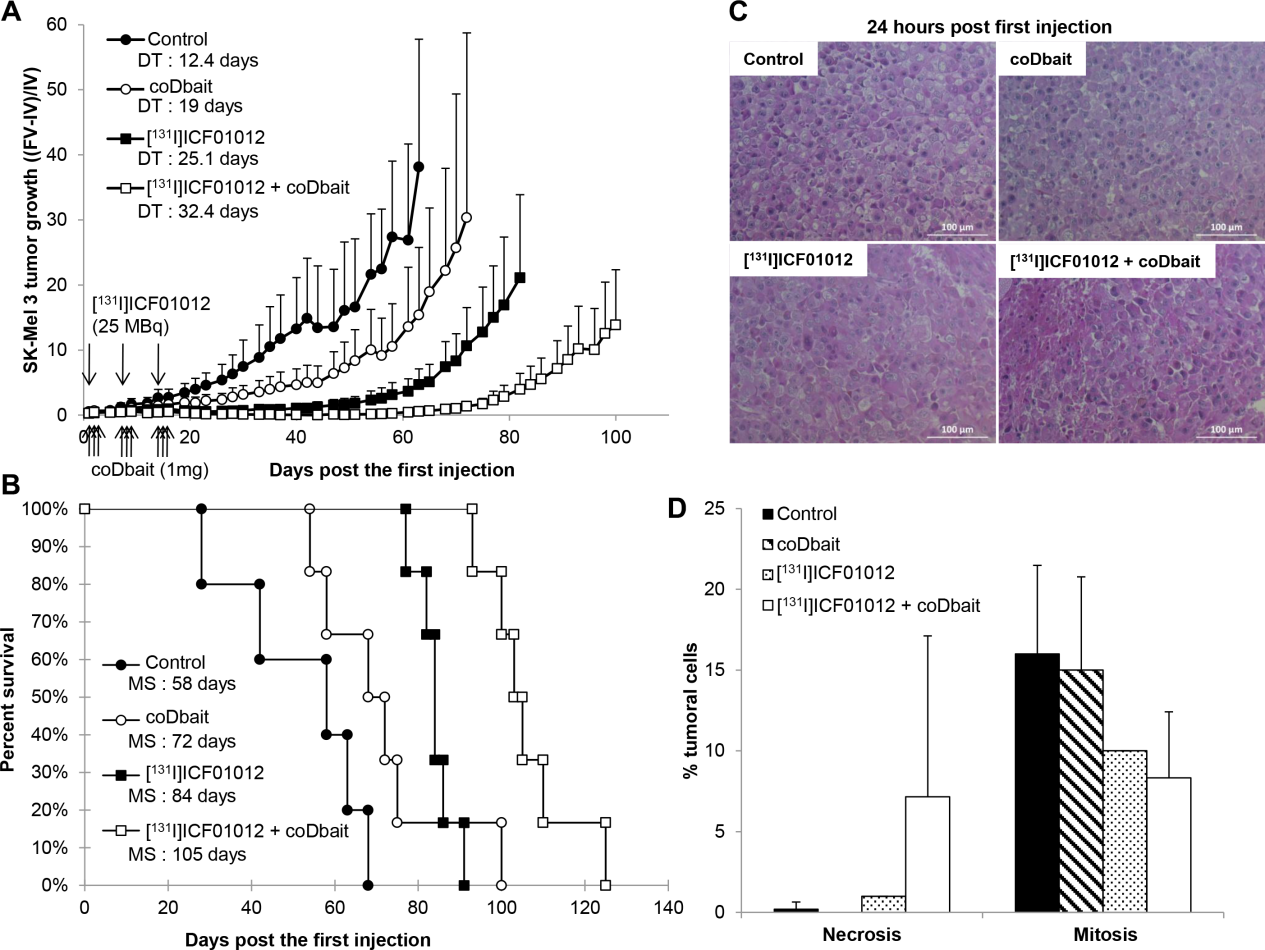
Effect of coDbait ± [^131^I]ICF01012 TRT in SK-Mel 3 model (**A**) The tumor growth was calculated with the ratio of final volume (FV) minus initial volume (IV) to initial volume (IV). The doubling time (DT) was calculated with the exponential value of tumor growth with six mice in each group. (**B**) Percentage survival of mice (*n* = 6 /group) receiving the different treatments. (**C**) HES representative histological sections and of SK-Mel 3 sampled 24 h after [^131^I]ICF01012 injection (25 MBq) and/or one injection of coDbait (1 mg). (**D**) The percentage of necrosis established by counting the cells on 3 HPF. *p < 0.05.

[^131^I]ICF01012 (3 × 25 MBq) injection in the SK-Mel 3 model produced a significant slowing in tumor progression compared with control group (doubling time: 12.4 *vs* 25.1 days, PHt, *p* = 0.006). In SK-Mel 3, the effect of [^131^I]ICF01012 was improved by coDbait, leading to slowed tumor growth (Figure 3A) (PHt, *p* = 0.06). The benefit of the association between TRT and coDbait was statistically supra-additive demonstrating a clear synergy of the two molecules on tumor growth control (comparison coDbait/combination: *p* < 0.001; comparison TRT/combination *p* = 0.005) (Supplementary Table 1B).

A statistically significant increase in the survival rate of mice receiving both compounds against single treatments was demonstrated (Cox, *p* = 0.005 and *p* = 0.017 compared with coDbait and [^131^I]ICF01012 respectively) (Figure 3B). In parallel to tumor growth, the association between TRT and coDbait was again statistically supra-additive demonstrating a clear synergy of the two molecules on mice survival (comparison coDbait/combination: *p* = 0.005; comparison TRT/combination *p* = 0.017) (Supplementary Table 1B). Tumor growth was controlled for 2 months before relapse. Double treatment was well tolerated; weight loss did not exceed 10% of the initial animal weight (Supplementary Figure 3A). Histology studies showed the emergence of cells with atypical nuclei and enlarged cytoplasm in tumors receiving [^131^I]ICF01012 ± coDbait (Figure 3C). In these groups necrosis was present in each animal and represented 20% in 2/5 mice with double treatment. The number of mitotic cells (< 10 mitosis per 10 high fields) decreased compared with control groups (> 15 mitosis per 10 high fields) (Figure 3D).

### *In vivo* antitumor mechanisms of [^131^I]ICF01012 + coDbait administration on B16Bl6 and SK-Mel 3 tumors

**Figure 4:**
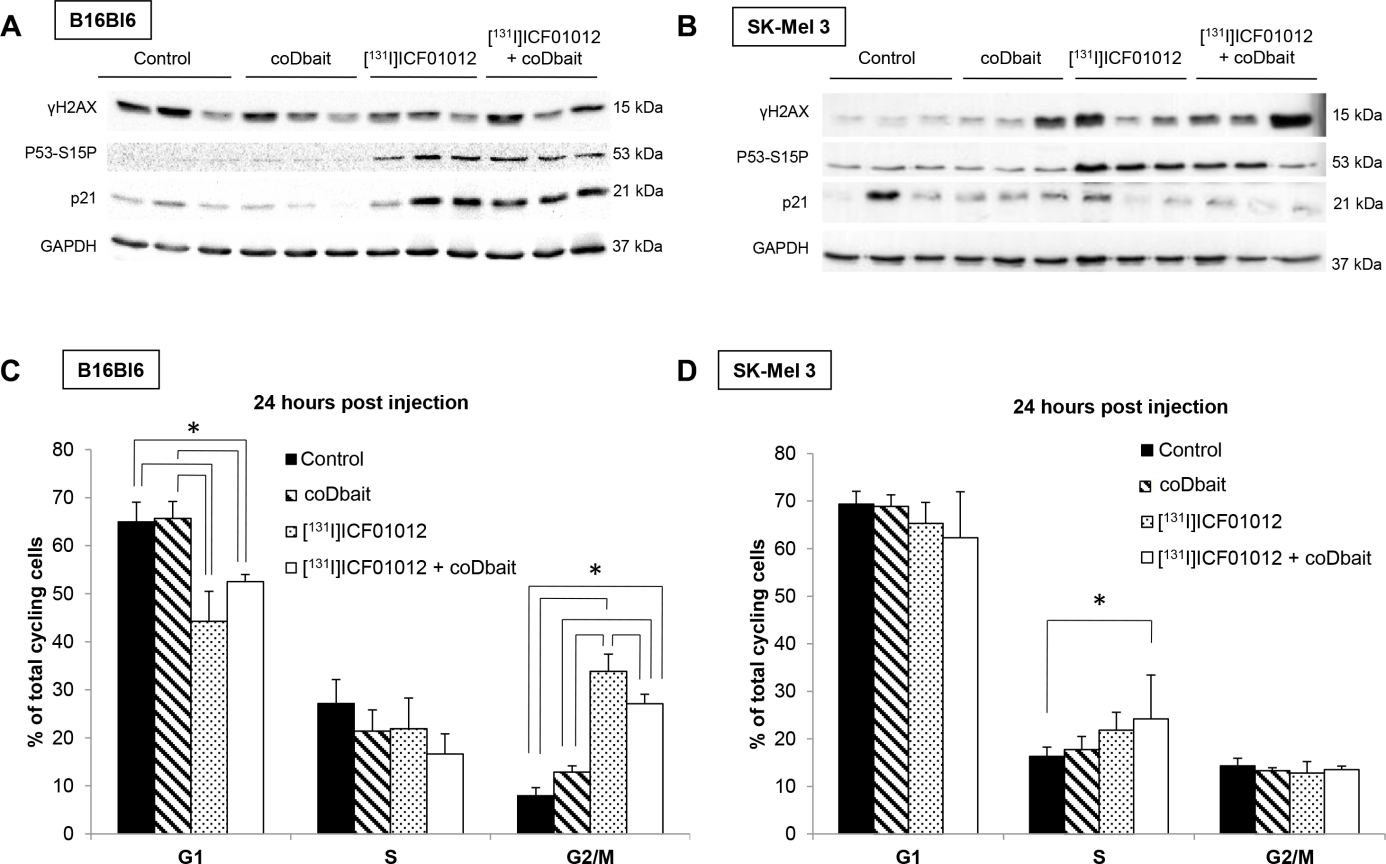
Mechanistic studies of [^131^I]ICF01012 ± coDbait on tumor extracts Western blot analysis of p53 phosphorylation (S15), ATM phosphorylation (S1981), Chk2 phosphorylation (T68) p21 expression and PARP cleavage in tumors 24 h p.i. in B16Bl6 (*n* = 3 tumors per group) (**A**) and SK-Mel 3 models (*n* = 3 tumors per group) (**B**). Cell cycle modifications induced 24 h after treatment in B16Bl6 (**C**) and SK-Mel 3 models (**D**).*p < 0.05.

[^131^I]ICF01012 TRT was expected to induce DNA double-strand breaks (DSB). We monitored the modifications (level and/or specific phosphorylation) 24 hours after irradiation of proteins involved in DNA repair and cell cycle control/death: γH2AX, P53-S15P and P21 in both models (Figure 4A and 4B). The basal γH2AX level was high in the B16Bl6 model with no significant modification observed in tumors receiving TRT and/or coDbait (Figure 4A). In contrast, γH2AX in SK-Mel 3 tumors increased significantly in TRT ± coDbait groups compared with the controls (Figure 4B). Moreover, the levels of P53-S15P were increased significantly in groups treated with [^131^I]ICF01012 in both models but without difference when groups received coDbait (Figure 4A and 4B). P53-S15P led to an induction of p21 expression only in the B16Bl6 model (Figure 4A).

We then analyzed cell cycle modifications (Figure 4C and 4D). In the P53 wild-type B16Bl6 tumors 24 h after treatment, a significant decrease in cells in G1 (44.3 ± 6.2% and 52.5 ± 1.5% *vs.* 64.9 ± 4.1% and 65.7 ± 3.5%) and an increase in G2/M cells (33.8 ± 3.5% and 27.1 ± 1.9% *vs.* 12.9 ± 1.2% and 7.9 ± 1.7%) were observed between groups receiving or not receiving [^131^I]ICF01012 (PH*t* test, *p* < 0.05). This pattern suggests that [^131^I]ICF01012 treated cells died from unrepaired damage that ultimately pass the G2/M checkpoint arrest leading to mitotic catastrophe in senescent or necrotic cells as previously reported [6]. Interestingly, coDbait addition significantly decreased G2/M blockade (PH*t* test, *p* < 0.05), suggesting that the disorganization of repair machinery could accelerate such cell death (Figure 4C). In contrast, in the human xenograft, 24 h following treatment, we did not observe a G2/M blockade regardless the treatment but a progressive increase of cells in S phase from control to double treated groups that became significant in the [^131^I]ICF01012 ± coDbait group compared with controls (Figure 4D). In SK-Mel3 model, slow-down of DNA replication induced decrease of tumor growth.

## DISCUSSION

The internal radiotherapy with [^131^I]ICF01012 is applicable on melanin-positive disseminated lesions. Using adapted dosimetry, tumor growth control and survival improvement can be observed after [^131^I]ICF01012 treatment in highly pigmented B16Bl6 tumors (18.5 MBq) and moderate SK-Mel 3 pigmented tumors (3 × 25 MBq) [6, 10]. Here, we demonstrated for the first time the possibility to increase [^131^I]ICF01012 TRT efficiency with the association of a pan DNA repair disruptor [17–19]. In both models, survival and tumor growth control were improved in mice receiving double treatment compared to those receiving single ones. This increase was shown to be additive for the B16Bl6 model and synergistic in SK-Mel 3 one. Assessment of radiosensitization required synergy demonstration, however the additive effects are also of clinical interest in front of the genetic heterogeneity observed in melanomas [24]. Different responses to TRT and to coDbait plus TRT are necessarily linked to variations in radioresistance mechanisms, *i.e*. signaling and repair. In both melanoma models, [^131^I]ICF01012 treatment resulted in DNA DSBs, leading to P53 phosphorylation on serine 15. In turn, the expression of P21 was induced in murine cells but not in human cells. Indeed in SK-Mel 3 the identified mutation (Arg267Trp) occurring in the DNA binding protein did not allow p21 transcription. On the contrary, in B16Bl6 tumors, P21 expression occurred and should block mitosis promoting factor activation (cyclin B1/CDk1; Chk1) [25] leading to mitotic giant cells. No G2/M nor G1/S blockade was seen in the SK-Mel 3 receiving [^131^I]ICF01012 (25 MBq). An important feature of human melanomas resides in a defective G2 checkpoint [13, 25] that could abrogate G2/M blockage in irradiated SK-Mel 3 tumors. P53 loss of function has been associated with slowing of S-phase following irradiation [26] as observed in SK-Mel 3 [^131^I]ICF01012 tumors. This phase S slow-down reached significance in the double treated group receiving one [^131^I]ICF01012 injection (25 MBq) and 1 mg of coDbait, this effect might be emphasized when treatment was completed, leading to significant tumor growth decrease and improved survival. Accumulation of cells in S-phase often reveals unrepaired damage that slows down the replication and may lead to cell death. These radiosensitizing effects seem comparable to those observed in preclinical models treated by EBR [17, 21]. However, the fine mechanisms implicated the DNA repair mechanisms following long ionizing radiation exposure with low dose rate need to be defined, peculiarly the involvement of homologous recombination towards non-homologous end joining [27]. The other important parameter in radioresistance is the ability of tumor cells to repair DNA lesions. We observed a high basal rate of γH2AX in B16Bl6 and to a lesser extent in SK-Mel 3 tumor controls. This result indicates the presence of spontaneous DNA breaks or replication stress [16, 28], and suggests active repair mechanisms. Inhibiting and/or disorganizing DNA repair system by coDbait clearly increased the effects of [^131^I]ICF01012 TRT in both models in regard to growth control and survival. In B16Bl6 melanomas, micronuclei that testify the lack of effective DNA repair were higher in double treated tumors.

The positive association of coDbait with TRT on melanoma whatever their p53 and b-raf status is of importance in treating patients with disseminated lesions, ineligible or resistant to available therapies. Another positive aspect for this combination is the absence of toxic effects after coDbait treatment. As demonstrated in the syngeneic B16 model, there is no modification of weight or a decrease in vital signs. The same applied to the hair follicle melanocytes, which were not altered in terms of number and morphology in groups receiving coDbait and/or [^131^I]ICF01012. However, the [^131^I]ICF01012 binding at the retina leads to a decrease of the layer thickness containing the photoreceptors just around the optic nerve. Importantly, the addition of coDbait does not increase the side effect even if there can readily diffuse in the body [23]. The impact of [^131^I]ICF01012 on retina must be contrasted. Indeed, a dosimetry performed in monkeys, with a similar molecule to [^131^I]ICF01012 [7], showed that the adsorbed dose in eye did not exceed the maximal tolerated dose. Moreover, in the clinical study of BA52 [8], no side effects on the retina was reported. In conclusion, the effect of [^131^I]ICF01012 is biased by a high melanin level on C57Bl6 mice eyes, that does not reflect the melanin content in humans eyes.

Although active therapeutics are now available to treat melanoma, i.e. kinase inhibitors and immune system modulators, they encounter resistance mechanisms [29]. The development of targeted therapies against intrinsic melanoma properties, such as the presence of melanins, offered another window for those patients without therapy proposal. Interestingly a phase I study with specific antibody (ab) against extracellular melanins radiolabelled with 188-rhenium showed the safety of this strategy and a survival improvement of patients with metastatic melanoma [30]. This strategy is currently being improved with the development of the corresponding humanized ab [30]. Another specific melanoma target (MSCP) was also challenged with ab radiolabelled with α particles emitters. In a clinical phase I study, anti-MSCP radiolabelled reported no toxicity and showed an increase of survival for some patients [31]. However, targeting tumors with radiolabelled antibodies suffers from their blood half life leading to hematopoietic toxicity and for their poor diffusion in solid tumors [32]. Small molecules such as ICF01012 can instead be delivered everywhere, rapidly eliminated and be retained specifically in pigmented organs. An ongoing clinical transfer for [^131^I]ICF01012 TRT with a first into human escalation dose phase will include patients with stage IV melanomas. The safety and efficiency of coDbait with EBR [33], already tested in patients with melanoma cutaneous recurrence, support the idea of future clinical combination with [^131^I]ICF01012 TRT.

## MATERIALS AND METHODS

### Cell culture and coDbait

Murine B16Bl6 and human SK-Mel 3 melanoma cell lines were obtained from the laboratory of Prof. Fidler (Houston, Texas, USA) and American Type Culture Collection (ATCC, Biovalley, Marne-La-Vallée) and were cultured as previously reported [10]. B16Bl6 is a cell line wild type for p53, b-raf and n-ras genes [34], while SK-Mel 3 harbors mutated P53 (Arg267Trp) and B-RAF (V600E) phenotypes [35, 36]. Dbait fused with cholesterol (coDbait) was from DNA Therapeutics (Evry, France) and corresponds to clinical DT01 [17].

### Radiolabelling of [^131^I]ICF01012

ICF01012 was labelled with [^131^I]NaI (881-1472 MBq, Perkin Elmer, Courtaboeuf, France) at high specific activity using a radioiododestannylation reaction as described elsewhere [5]. [^131^I]ICF01012 was obtained with good radiochemical yields (60–87%), high radiochemical purities (> 98%) and specific activity in the range 57–115 TBq/mmol.

### Tumor models and schedule treatment

Male C57BL/6J and female Nude mice (6 weeks old) were obtained from Charles River Laboratories (L'Abresle, France). All animal studies were carried out in accordance with the Guide for the Care and Use of Laboratory Animals and approved by the ethics committee (C2E2A, number CE64-12). To establish the tumors, 3 × 10^5^ B16Bl6 cells (C57BL/6J mice) and 5 × 10^6^ SK-Mel 3 cells (Nude mice) in 100 μL PBS buffer were injected subcutaneously into the right flank. Mice were i.v. injected with [^131^I]ICF01012 after 10 days for B16Bl6 (mean tumor volume: 30–100 mm^3^) and 35 days for SK-Mel 3 (mean tumor volume: 30–200 mm^3^) (Supplementary Figure 3B and 3C). The length and width of the tumors were measured using a caliper. Tumor volume was determined with the formula: volume (mm^3^) = (length (mm) × width^2^ (mm))/2. Mice were weighed three times per week and sacrificed when tumors reached 2000 mm^3^. This endpoint was considered as day of death in survival analyses.

After 10 days and 35 days of tumors growth, [^131^I]ICF01012 treatment was administered intravenously in mice bearing B16Bl6 (18.75 MBq, day 0) and SK-Mel 3 (3 × 25 MBq, days 0, 7 and 14) melanomas, respectively. Two independent experiments with 10 mice per group were performed for the B16Bl6 model, and one experiment with 6 mice per group for the SK-Mel 3 model. In both, coDbait diluted in 5% glucose was injected into the tumor and in the peritumoral area 5 h before each TRT (2 mg and 1 mg for B16F6 and SK-Mel3, respectively). Further injections of coDbait were made at days 1, 4, 6, 8 after TRT for B16Bl6 (2 mg per injection) or at 24 h and 48 h after each radiotherapy for the SK-Mel3 model (1 mg per injection). Controls received 5% glucose solution in the same manner. For molecular studies, three additional mice per group were killed 24 h and/or 10 days post-irradiation. Tumors were removed and divided into two parts, one fixed in AFA for histology and one frozen in liquid N_2_. Metastases were counted on lung using a binocular microscope.

### Histological analyses

Histological (tumors and eyes) and cell cycle analyses were performed as previously described [6]. Micronuclei assessment was performed on HES staining as previously described [17], approximately 3 500 cells per groups have been counted.

Eyes were placed in fixative solution and embedded in paraffin. Sections of 5 μm were cut and prepared for haematoxylin-eosin saffron staining. The thickness of the retina pigment epithelium and that of photoreceptor were measured both near to and far from the optic nerve using MetaMorph software from the CICS platform (Clermont-Ferrand, France).

### Molecular studies

For Western blot, proteins were extracted from 10 mg of crushed tumor in buffer (6 M urea, 5 mM NaF, 2.5 mM sodium pyrophosphate, 1 mM EDTA, 0.5% TritonX100, 1 mM activated sodium orthovanadate, 1X protease inhibitors). Proteins (30 μg) were separated in 8% or 12.5% SDS-PAGE and transferred to nitrocellulose membranes (Millipore, St Quentin-en-Yvelines, France). We used the following antibodies: p53-S15P (9284, Cell Signalling, Ozyme, St-Quentin-en-Yvelines, France), γH2AX (2577, Cell Signalling), p21 (6246, Santa Cruz, Clinisciences, Nanterre, France), and GAPDH (25778, Santa Cruz). VEGF level was determined by ELISA (Raybiotech, Tebu-bio SA, Le Perray-en-Yvelines, France) according to the manufacturer's instructions.

### Statistical analysis

The results are expressed as mean ± SD except for Figures 1A and 3A where they are represented as mean ± SEM. Statistical analyses were conducted using Stata software (version 13, StataCorp, College Station, TX, US). A two-tailed *p* value of less than 0.05 was considered to indicate statistical significance. Comparisons concerning quantitative parameters (tumor doubling time, VEGF content, lung metastases distribution) between treatment groups were made using ANOVA or a non-parametric Kruskal-Wallis test according to ANOVA hypotheses (assumption of normality studied by Shapiro-Wilk and homoscedasticity by Bartlett's test). When appropriate (previous tests such as *p* < 0.05), an appropriate *post hoc* test (PHt) was considered: Tukey-Kramer (TK) followed ANOVA and Dunn (D) for Kruskal-Wallis. Finally, for the analysis of repeated measures (tumour growth), the random effects model (REM) was considered, as usually proposed, to study the fixed effects treatment groups, time-points and their interaction group × time taking into account between- and within-subject variability. Censored data (survival) were estimated using the Kaplan-Meier method. The log-rank test was used in univariate analysis to compare independent groups. The results were expressed as hazard ratios (HR) after Cox proportional hazards regression (Cox). The probability of the endpoint (death, or any other event of interest, e.g. recurrence of disease) is called the hazard. The hazard is modeled as:

$$\text{H}(t)={\text{H}}_{0}(t)\times \text{exp}(b_{1}X_{1}+b_{2}X_{2}+b_{3}X_{3}+\ldots +b_{k}X_{k})$$

where X_1_ … X_k_ are a collection of predictor variables and H_0_ (t) is the baseline hazard at time *t*, representing the hazard for a person with the value 0 for all the predictor variables.

By dividing both sides of the above equation by H_0_ (t) and taking logarithms, we obtain:

$$\text{In}\left(\frac{\text{H}(t)}{{\text{H}}_{0}(t)}\right)=b_{1}X_{1}+b_{2}X_{2}+b_{3}X_{3}+\ldots +b_{k}X_{k}$$

We call H (t) / H_0_ (t) the hazard ratio. The coefficients b_i_…b_k_ are estimated by Cox regression, and can be interpreted in a similar manner to that of multiple logistic regression.

The type of interaction was additive when corresponding to the effect (b_i_…b_k_) being equal to that of the theoretically calculated effects of [^131^I]ICF01012 or coDbait alone and supra-additive (synergistic) when the effect of combined [^131^I]ICF01012 plus coDbait is considered to be more efficient than the calculated effect of single use. The term synergy is considered to correspond to supra-additivity [37]. The same reasoning can be applied to random-effects models issues.

## SUPPLEMENTARY MATERIALS FIGURES AND TABLE


