# Supplementary material for: Targeting DNA repair by coDbait enhances melanoma targeted radionuclide therapy
Source: Oncotarget. 2016 Feb 12;7(11):12927–36. doi: 10.18632/oncotarget.7340 (PMC4914332; doi:10.18632/oncotarget.7340)
Supplement: Supplementary file 1 [file oncotarget-07-12927-s001.pdf]

Targeting DNA repair by coDbait enhances melanoma targeted radionuclide therapy

Supplementary Materials

A

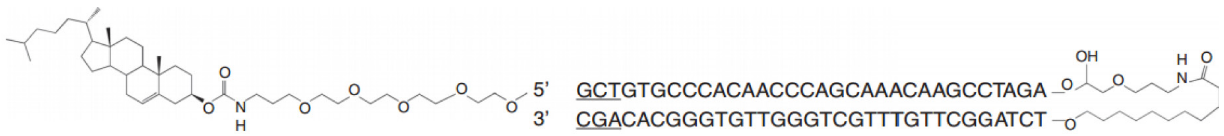

B

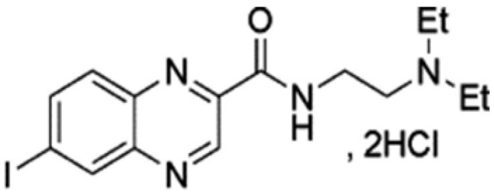

Supplementary Figure S1: Structures of coDbait (A) and ICF01012 (B).

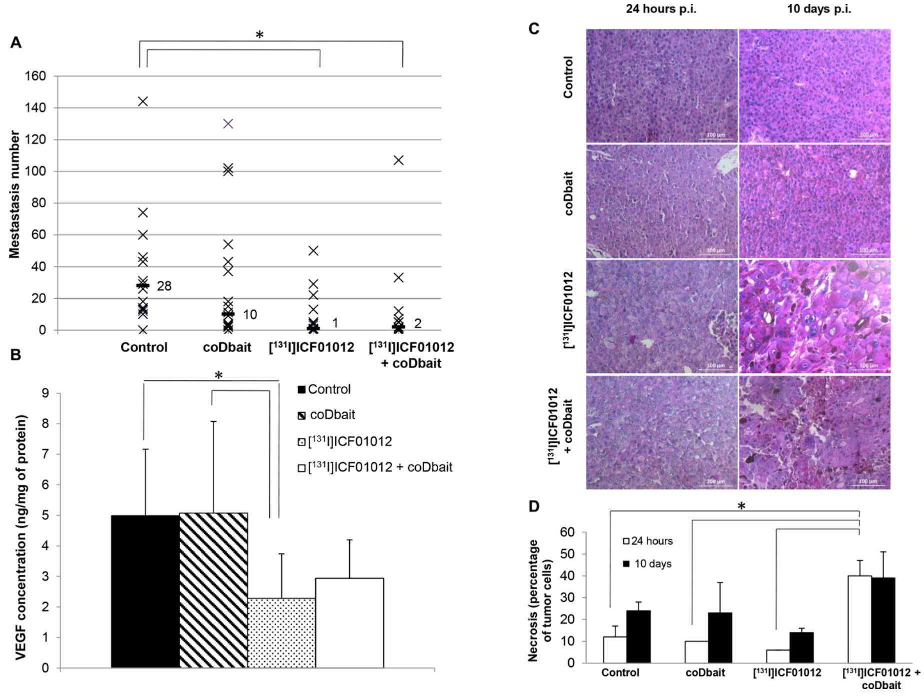

Supplementary Figure S2: Effects of combined treatment on B16Bl6 dissemination and anatomopathologic features. (**A**) The number of lung metastases was significantly decreased in mice receiving [^{131}I]ICF01012 ± coDbait (n = 15 each) compared with control (n = 14) and coDbait (n = 18) groups. (**B**) Tumor VEGF content was decreased significantly in [^{131}I]ICF01012 treatment compared with control and coDbait. (**C**) HPS representative histological sections of B16Bl6 tumors 24 h or 10 days post-[^{131}I]ICF01012 irradiation with one dose of coDbait (for 24 h, 2 mg) or with complete treatment (10 days, 5 × 2 mg). 24 h post-irradiation, tumor cells were larger, with a marked nucleolus in both groups, with and without coDbait. An atypical population of giant cells with expanded cytoplasm and sharpened nucleoli in the TRT group was observed 10 days post-[^{131}I]ICF01012 injection in all samples, representing 10–20% of the tumor cells (**D**) The percentage of necrosis was established by counting the cells on 3 HPF. *$p < 0.05$.

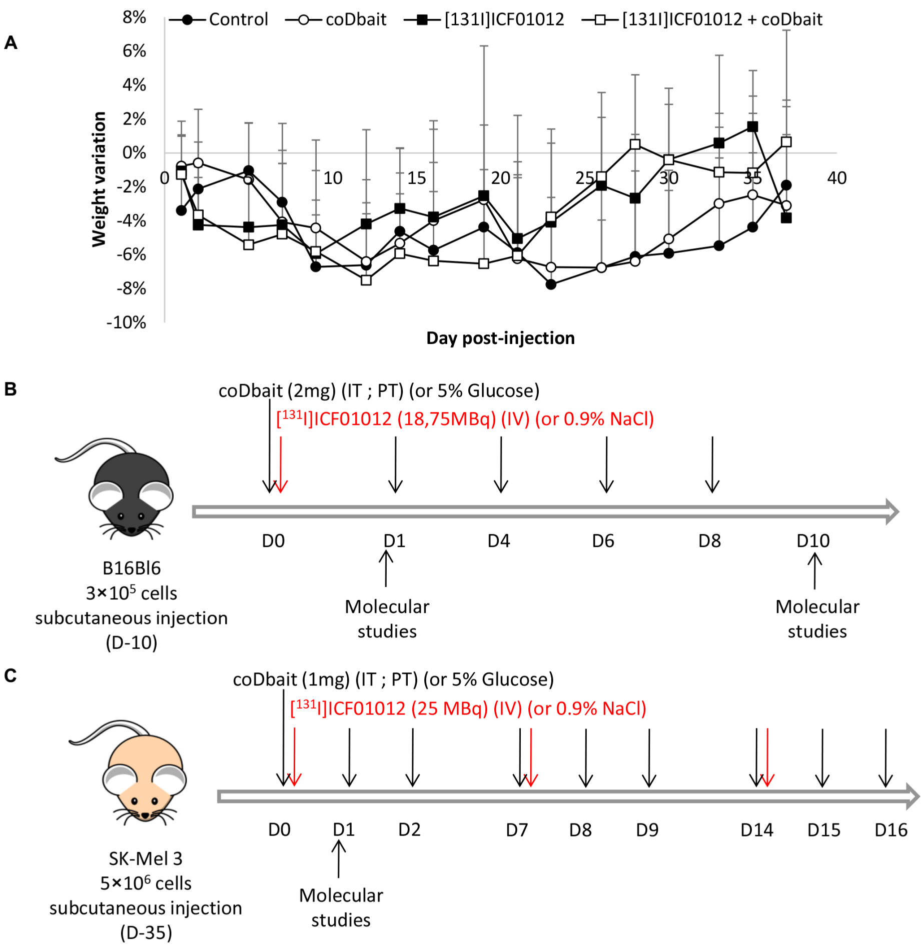

Supplementary Figure S3: (**A**) Effect of coDbait ± [^{131}I]ICF01012 TRT on the mouse weight in SK-Mel 3 model, the results are expressed as the variation from the initial value, the bars represent the SD ($n = 6$ per group). Schedules of experimental *in vivo* radiotherapy protocols in B16Bl6 (**B**) and SK-Mel 3 models (**C**).

Supplementary Table S1: Detailed statistical analyses of tumor volume and survival of both melanoma models

A) B16Bl6 MODEL

Tumor volume

Groups	Coef.	Std. Err.	z	*p*-value	[95% Conf. Interval]	
Control	REFERENCE					
coDbait	−266.5966	80.03452	−3.33	0.001	−423.4614	−109.7319
[131I]ICF01012	−532.2799	81.80356	−6.51	0.000	−692.6119	−371.9479
[131I]ICF01012 + coDbait	−649.0953	81.6609	−7.95	0.000	−809.1477	−489.0428

$p < 0.001$ (coDbait *vs.* [131I]ICF01012 + coDbait)
$p = 0.26$ ([131I]ICF01012 *vs.* [131I]ICF01012 + coDbait)
266.60 + 535.28 = 798.88 > 649.10
The coefficients (Coef.) correspond to the slope values.

Survival

Groups	Coef.	Std. Err.	z	*p*-value	[95% Conf. Interval]	
Control	REFERENCE					
coDbait	−1.992727	.4596066	−4.34	0.000	−2.893539	−1.091914
[131I]ICF01012	−2.422825	.4913346	−6.51	0.000	−3.385823	−1.459827
[131I]ICF01012 + coDbait	−3.129921	.5103184	−6.13	0.000	−4.130127	−2.129715

$p = 0.005$ (coDbait *vs.* [131I]ICF01012 + coDbait)
$p = 0.06$ ([131I]ICF01012 *vs.* [131I]ICF01012 + coDbait)
1.99 + 2.42 = 4.41 > 3.13

B) SK-MEL 3 MODEL

Tumor volume

Groups	Coef.	Std. Err.	z	*p*-value	[95% Conf. Interval]	
Control	REFERENCE					
coDbait	−196.2967	107.1558	−1.83	0.067	−406.3182	13.72486
[131I]ICF01012	−437.3487	106.9216	−4.09	0.000	−646.9112	−227.7862
[131I]ICF01012 + coDbait	−734.4674	106.8752	−6.87	0.000	−943.939	−524.9958

$p < 0.001$ (coDbait vs. [131I]ICF01012 + coDbait)
$p = 0.005$ ([131I]ICF01012 vs. [131I]ICF01012 + coDbait)
196.3 + 437.3 = 633.6 < 734.5

Survival

Groups	Coef.	Std. Err.	z	*p*-value	[95% Conf. Interval]	
Control	REFERENCE					
coDbait	−1.915589	.8082333	−2.37	0.018	−3.499697	−.3314804
[^{131}I]ICF01012	−2.300733	.8331452	−2.76	0.006	−3.933668	−.6677984
[^{131}I]ICF01012 + coDbait	−4.436259	1.064646	−4.17	0.000	−6.522927	−2.34959

$p = 0.005$ (coDbait vs. [^{131}I]ICF01012 + coDbait)
$p = 0.017$ ([^{131}I]ICF01012 vs. [^{131}I]ICF01012 + coDbait)
1.92 + 2.30 = 4.22 < 4.44

The Coef. for both single treatments were added. The sum was compared to the slope value of the combined treatment and if there was superior to the sum, the effect was syngergic (calculs in red, under the table).
